# Ursodeoxycholic acid augmentation in treatment-refractory schizophrenia: a case report

**DOI:** 10.1186/s13256-020-02484-9

**Published:** 2020-09-01

**Authors:** Mohsen Khosravi

**Affiliations:** Department of Psychiatry and Clinical Psychology, Zahedan University of Medical Sciences, Zahedan, 9813913777 Iran

**Keywords:** Schizophrenia, Therapeutics, Ursodeoxycholic acid, Report

## Abstract

**Background:**

Treatment-resistance is recognized as a significant dilemma in schizophrenia, which has been reported to involve approximately one-third of patients with schizophrenia.

**Case presentation:**

This case report described a 12-week treatment course for a 39-year-old Persian man with treatment-refractory schizophrenia, who showed a significant improvement in terms of positive, negative, and cognitive symptoms after taking ursodeoxycholic acid 300 mg capsules twice a day. Also, ursodeoxycholic acid was well tolerated, and he did not exhibit any side effects during treatment, based on interview and physical examination.

**Conclusion:**

Ursodeoxycholic acid augmentation seems to be an effective treatment strategy for patients with treatment-refractory schizophrenia. However, further investigations in this field need to be carried out through randomized controlled trials.

## Background

Treatment-resistance is a significant dilemma in schizophrenia, which has involved many patients, their families, and health care professionals. Treatment-refractory schizophrenia (TRS) is defined as a treatment failure despite the use of two adequate treatment trials with antipsychotics of two different classes (including a 4-to-6-week trial of 400 to 600 mg/day chlorpromazine or its equivalent). TRS affects approximately one-third of patients with schizophrenia [1]. Over the past decade, many clinical practice guidelines have recommended clozapine for patients with TRS; however, only 40% of the patients have met the response criteria [2]. Furthermore, there has been no convincing evidence for the augmentation of clozapine yet; Even “novel” therapies, including N-methyl-D-aspartate receptor (NMDAR) enhancers (such as glycine, D-serine, D-cycloserine, and N-methylglycine), yielded contradictory results in clinical trials [3].

Bile acids are known as an associated category of molecules extracted from cholesterol and have been used as therapeutic agents in medicine for a long time [4]. Recent studies have shown that ursodeoxycholic acid (UDCA) and its main conjugate, that is, glycoursodeoxycholic acid (GUDCA), are bile acids with neuroprotective and homeostatic properties due to their capacity to inhibit glutamate release [5]. Based on these findings, a possible hypothesis is presented, stating that UDCA may be effective in reducing the psychotic and cognitive symptoms among patients with schizophrenia. In line with the above hypothesis, our report presented a 12-week treatment course for a patient with TRS who made significant improvement in positive, negative, and cognitive symptoms following initiation of UDCA.

## Case presentation

**Fig. 1:**
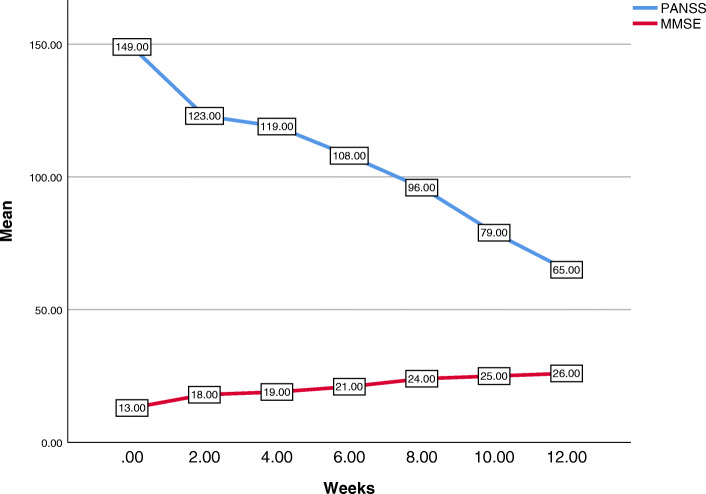
Multiple-line mean of Mini-Mental State Examination and Positive and Negative Syndrome Scale over weeks. *MMSE* Mini-Mental State Examination, *PANSS* Positive and Negative Syndrome Scale

A 39-year-old Persian man, without any positive family history, was referred to our hospital with a 15-year history of command hallucination, persecutory delusion, social isolation, hostility/excitement, anxiety, depression, and cognitive impairment, which had been treated at a psychiatric clinic with the diagnosis of schizophrenia. Although he had already been admitted to a psychiatric hospital six times during 15 years, his records indicated the progressive course of the disease and his failure to respond to various treatment regimens. No abnormal findings were observed in a physical examination, imaging, and laboratory tests. Finally, he was treated with clozapine with a diagnosis of TRS, which was eventually terminated due to its adverse effects, including restlessness, tachycardia, tremor, sialorrhea, nausea, and disturbed sleep. After discontinuation of clozapine, although he was treated with olanzapine, carbamazepine, fluvoxamine, lorazepam, propranolol, and fluphenazine decanoate injection for 2 years, no significant improvement was made. At the end of the second year of treatment with the above medication regimen, mirtazapine of 15 mg/day was added to the previous regimen and increased to 30 mg/day due to exacerbation of anorexia, insomnia, and anxiety. Despite a relative improvement in anorexia, anxiety, and insomnia, a laboratory test revealed an elevated alanine transaminase (ALT) level compared to the previous test (147 U/L versus 22 U/L; reference range, up to 41 U/L). Given an internal medicine consultation, mirtazapine was discontinued after 2 months, and he started taking UDCA 300 mg capsules twice a day. In the fourth week of treatment, his ALT level was reduced (28 U/L; reference range, up to 41 U/L). Also, his positive, negative, and cognitive symptoms significantly decreased during the eighth week of follow-up and continued with a mild slope throughout the tenth and 12th weeks of the treatment. There was a significant difference between the mean scores of the Positive and Negative Syndrome Scale (PANSS) and the Mini-Mental State Examination (MMSE) before and 12 weeks after the treatment with UDCA (149 versus 65 and 13 versus 26, respectively). Also, UDCA was well tolerated, and our patient did not exhibit any side effects during treatment based on interview and physical examination. Figure 1 presents the 12-week follow-up of our patient according to PANSS and MMSE.

## Discussion and conclusions

Although the dopamine hypothesis of schizophrenia has been raised, poor response to antipsychotics may indicate that some patients with TRS have suffered from non-dopamine pathophysiology [6]. In this respect, a theory that was first proposed in the 1980s, and gained further support in the following years, attributed the biological origin of schizophrenia symptoms to the glutamate neurotransmitter, rather than dopamine [7]. This theory states that NMDAR dysfunction in schizophrenia leads to excessive glutamate release of cortico-pyramidal neurons, which can produce the full range of schizophrenia symptoms and cognitive impairments [6].

Consistent with this theory, new research over the past decade has suggested that bile acids may reduce the production of glutamate [5]. For instance, Silva *et al.* [5] claimed that the overstimulation of glutamate receptors to induce excitotoxicity in neurons isolated from late-stage fetal rat brains was largely suppressed by GUDCA. As a result, the bile acid seems to inhibit glutamate release in either normal or microglia-depleted hippocampal tissue slices.

Our results demonstrated that UDCA augmentation might be an effective treatment strategy for patients with TRS. According to the glutamate hypothesis of schizophrenia models, some of the possible therapeutic mechanisms are: (a) A reduced tone of glutaminergic projection neurons —which under-stimulates GABAergic interneurons in the ventral tegmental area— might lead the mesocortical dopamine pathway to be activated. This procedure results in sufficient dopamine release in the prefrontal cortex (PFC) and, in turn, decreases the cognitive and negative symptoms [8]; (b) the reduced firing of cortical glutaminergic projection neurons could cause dopamine mesolimbic pathway hypoactivation, which diminishes the positive symptoms [8, 9].

Moreover, retinoic acid as a neurotrophic factor shares a common role in regulating lipid homeostasis with bile acids via activating the farnesoid X receptor (FXR) signaling pathway or vice versa. Accordingly, bile acids are likely to exert mediating effects on the nervous system through a similar mechanism that is not completely known [4, 10].

Although the clinical consumption of UDCA has been acceptably safe, the UDCA efficacy for TRS is still debated. The molecular functions of bile acids have been widely discovered; however, the mechanism of UDCA needs to be further explored. Further studies on bile acids may be beneficial for developing more efficient approaches to using this classical compound as a new drug in TRS.

## Data Availability

The patient’s information and medical records used for the case report are available from the corresponding author upon request.

## Abbreviations

ALT: Alanine transaminase
FXR: Farnesoid X receptor
GUDCA: Glycoursodeoxycholic acid
MMSE: Mini-Mental State Examination
NMDAR: N-methyl-D-aspartate receptor
PANSS: Positive and Negative Syndrome Scale
PFC: Prefrontal cortex
TRS: Treatment-refractory schizophrenia
UDCA: Ursodeoxycholic acid
